# Do method and species lifestyle affect measures of maximum metabolic rate in fishes?

**DOI:** 10.1111/jfb.13195

**Published:** 2016-10-25

**Authors:** S. S. Killen, T. Norin, L. G. Halsey

**Affiliations:** ^1^ Institute of Biodiversity, Animal Health and Comparative Medicine, Graham Kerr BuildingUniversity of GlasgowGlasgowG12 8QQU.K.; ^2^ Department of Life SciencesUniversity of RoehamptonHolybourne AvenueLondonSW15 4JDU.K.

**Keywords:** aerobic scope, ecophysiology, fishes, locomotion, metabolism, teleosts

## Abstract

The rate at which active animals can expend energy is limited by their maximum aerobic metabolic rate (MMR). Two methods are commonly used to estimate MMR as oxygen uptake in fishes, namely during prolonged swimming or immediately following brief exhaustive exercise, but it is unclear whether they return different estimates of MMR or whether their effectiveness for estimating MMR varies among species with different lifestyles. A broad comparative analysis of MMR data from 121 fish species revealed little evidence of different results between the two methods, either for fishes in general or for species of benthic, benthopelagic or pelagic lifestyles.

The maximum aerobic metabolic rate (MMR) of an animal imposes an upper constraint on the capacity for performing oxygen‐consuming physiological activities. This capacity is the aerobic scope, which is the difference between the animal's MMR and its standard (minimum) metabolic rate (SMR) and is the range of metabolic rates within which the animal must function. There has been a growing focus on understanding how MMR may influence behaviour at the inter and intraspecific levels, with fishes being important model species in much of this research (Killen *et al.*, 2012, 2016; Metcalfe *et al.*, 2016; Norin & Clark, 2016). In addition, it has been hypothesized that MMR may be related to the ability of fish species to cope with climate change since the thermal performance curve for MMR may differ from that for SMR, thereby shaping aerobic scope differently across temperatures (Fry, 1971; Pörtner & Farrell, 2008; Clark *et al.*, 2013).

Coupled with this increasing interest in the relevance of MMR for ecology and conservation, there have been discussions about how best to estimate MMR of fishes in an experimental setting (Reidy *et al.*, 1995; Roche *et al.*, 2013; Norin & Clark, 2016). The most commonly used methods involve either a stepwise, ramped‐speed swimming protocol in a flume respirometer (swimming respirometry), or manually chasing the fish to exhaustion in a container, followed by immediate transfer to a static‐water respirometer or, in some cases, exhausting the fish in a flume and then transferring it to the static respirometer (post‐exercise respirometry). The key difference between these two methods is that the swimming respirometry method involves measurements of oxygen consumption rate $\left({\dot{M}}_{O_{2}}\right)$ during mostly steady‐state swimming, whereas the post‐exercise respirometry method involves measuring ${\dot{M}}_{O_{2}}$ immediately after exhaustive (burst‐type) swimming. The latter assumes that maximum ${\dot{M}}_{O_{2}}$ (*i*.*e*. MMR) occurs during an aerobic recovery phase following largely anaerobic exercise [see Norin & Clark (2016) for description of both techniques].

Both of these methods have potential limitations. For example, the swimming respirometry method usually allows the experimenter to measure only one or two fish per day because of the duration of the protocol and multiple flume respirometers are typically prohibitively expensive. In contrast, the post‐exercise respirometry method allows the researcher to obtain measurements much more quickly, particularly when using several (relatively inexpensive) respirometers in parallel on an intermittent‐flow setup (Svendsen *et al.*, 2016). For this reason, the post‐exercise respirometry method has become particularly attractive as more researchers study among‐individual variation in metabolic rates, which requires a relatively large number of fish to be tested. Both methods face the potential challenge of animal motivation; some individuals can be reluctant to swim against a water current within a flume and may stop swimming before they have reached their maximum potential swimming speed (Peake & Farrell, 2004, 2006). This could be caused by differences in the progressive transition to use of anaerobic metabolism to fuel swimming in a ramped‐speed swim challenge (Lee *et al.*, 2003; Svendsen *et al.*, 2010; Farrell, 2016). Similarly, fishes being chased or forced to swim in a post‐exercise protocol may also cease swimming before reaching the maximal rate at which oxygen can be transported to the tissues. Furthermore, whether measures of ${\dot{M}}_{O_{2}}$ during intense activity are equivalent to such measures during the recovery from intense activity has been questioned (Scott, 2005). In one species, the barramundi *Lates calcarifer* (Bloch 1790), ${\dot{M}}_{O_{2}}$ was found to be significantly higher immediately after, rather than during, an exhaustive chase (Norin & Clark, 2016).

Despite the wide interest in obtaining measures of MMR for advancing understanding of ecophysiology (Lefevre, 2016; Metcalfe *et al.*, 2016), it still remains unclear whether the estimates of MMR obtained from swimming and post‐exercise respirometry concur. Few studies have compared the two methods directly and those that have usually examined differences in a single species (Reidy *et al.*, 1995; Roche *et al.*, 2013), or at most a small number (Rummer *et al.*, 2016). It has also recently been proposed that morphology or lifestyle may cause some species to be more or less suitable for a particular method of measuring MMR (Norin & Clark, 2016). For example, more pelagic species that can be strong endurance swimmers may display a higher MMR when tested using the swimming respirometry method, whereas more benthic species that do not routinely engage in prolonged swimming may be more amenable to the post‐exercise respirometry method. A comparative approach based on the published literature was used to investigate whether the estimates of MMR obtained from swimming and post‐exercise respirometry methods concur and whether species lifestyle influences the measures of MMR obtained from the methods.

Data on MMR, estimated from measurements of ${\dot{M}}_{O_{2}}$, were collected from the literature for 121 species of teleosts. Measurements were included from studies that measured MMR either during peak levels of forced swimming *via* the swimming respirometry method, or immediately following exhaustive exercise in either a swim flume or by manual chasing, *i*.*e*. the post‐exercise respirometry method. Of the 121 species, there were 14 for which MMR data were available for both the swimming and post‐exercise respirometry methods (*i*.*e*. a total of 128 estimates of MMR; Table S1, Supporting Information). Classifications of species lifestyle were obtained from FishBase (Froese & Pauly, 2008), as either pelagic, benthopelagic or benthic. Pelagic species are those that live in the water column; benthopelagic species live and feed near, but not on, the substratum (including coral reefs), sometimes associating with mid‐waters or even surface waters depending on depth; benthic species live on the bottom, generally in contact with or just above the substratum. In general, pelagic species tend to be more active, constantly swimming species; benthic species tend to be more sedentary or sluggish; benthopelagic species tend to show intermediate levels of routine activity. For comparisons of method and lifestyle, only one species per lifestyle per method was used, to avoid giving undue weight to species represented by multiple studies (Killen *et al.*, 2010). When multiple datasets were available for a species, priority was given to measures performed within the natural temperature range of a species, but closest to 15° C (to minimize the range of temperatures included in the dataset). If multiple studies for a species performed measures at 15° C, the dataset with the largest mean body mass was used.

All analyses were performed with SPSS statistics 20.0 (SPSS Inc. and IBM; www.ibm.com/analytics/us/en/technology/spss/spss.html). The level of significance for all tests was *α* = 0·05. MMR and body mass were log_10_‐transformed to comply with assumptions of homogeneity of variance and normality. The overall effect of method on estimates of MMR (mg O_2_ h^−1^) was investigated using a general linear model (GLM) with method as a categorical variable and temperature and body mass as covariates. To further investigate the effects of lifestyle, a second GLM was run using only the data for the benthic and benthopelagic species (the pelagic category had a small sample size with only *n* = 4 for the post‐exercise method). This model had lifestyle and method as categorical variables, temperature and body mass as covariates and included an interaction between lifestyle and method. Data were also analysed with phylogenetic information, but with fewer species (*i*.*e*. only those for which phylogenetic information is available). For the latter, the phylogenetic generalized least squares (PGLS) method (Grafen, 1989; Martins & Hansen, 1997; Garland & Ives, 2000) was employed *via* the ape package (Paradis *et al.*, 2004) in R (www.r‐project.org), applying a phylogeny generated from the comprehensive tree of life (Hinchliff *et al.*, 2015) using the rotl package in R (Michonneau *et al.*, 2016), which was then manually augmented. The branch lengths were estimated using Grafen's branch‐length transformation (Grafen, 1989), where branch lengths were set to a length equal to the number of descendant tips minus one (Fig. S1, Supporting Information). For ease of visual inspection and representation in figures, MMR data for all species are presented adjusted for the effects of body mass (*M*, g) and temperature (*T*, ° C) using residuals from the following regression derived from the entire dataset (where MMR = *R*_MM_): log_10_ *R*_MM_ = −0·7377 + 0·9561 log_10_ *M* + 0·0233 *T* + *ϵ*; *r* ^2^ = 0·896, *P* < 0·001. Residuals were added to the fitted model values for *M* = 1000 g and *T* = 20° C (the mean temperature for all studies included in the dataset) to obtain standardized values for MMR. For the 14 species which had been measured for MMR using both the post‐exercise and swimming respirometry methods, the relationship between the values collected *via* each method across species was examined using simple linear regression.

Overall, the model outputs, both with and without accounting for phylogenetic inter‐relatedness, indicated no differences in estimates of MMR between the post‐exercise and swimming respirometry methods when considering the dataset as a whole [GLM, effect of method, *F*_1,124_ = 0·284, *P* > 0·05; Fig. 1(a)]. To examine how the effect of method may differ between lifestyles, based on sample sizes the most reliable comparison between methods could be made between benthic and benthopelagic species. There was a marginally significant interaction between lifestyle and method [GLM with benthic and benthopelagic data, effect of method × lifestyle, *F*_1,104_ = 3·998, *P* < 0·05; Fig. 1(b)] with estimates of MMR obtained *via* the post‐exercise method for benthopelagic species being higher than that of the benthic species. The difference was corroborated by comparisons of standardized post‐exercise MMR values between the benthic and benthopelagic species [Welch two‐sample *t*‐test, *t* = −3·987, d.f. *=* 36·27, *P* < 0·001; Fig. 1(b)]. This probably reflects a bias by which the most sluggish species (*e*.*g*. the most inactive benthic species), with correspondingly low MMR, can only be measured by the post‐exercise method owing to lack of motivation for continuous swimming. There was no main effect of method when lifestyle was included in the analysis (GLM, *F*_1,104_ = 0·027, *P* > 0·05). Only four pelagic species have been measured using the post‐exercise method. Although this precludes a statistical comparison, visual inspection reveals a large difference in MMR between the swimming and post‐exercise respirometry methods for this lifestyle [Fig. 1(b)]. It is worth noting, however, that the swimming method data for pelagic fishes include all of the high‐performance scombrid species (mackerels and tunas) with extremely high measures of MMR. Removal of the scombrids from the dataset produces more similar estimates of MMR between methods [Fig. 1(b)]. This observation relates to a larger confounding issue whereby, within any given lifestyle, relatively athletic species tend to be measured using the swimming respirometry method while less active species tend to be measured using the post‐exercise respirometry method. This should bias mean MMR values for the swimming respirometry method to be higher, while means for the post‐exercise respirometry method will tend to be lower.

**Figure 1:**
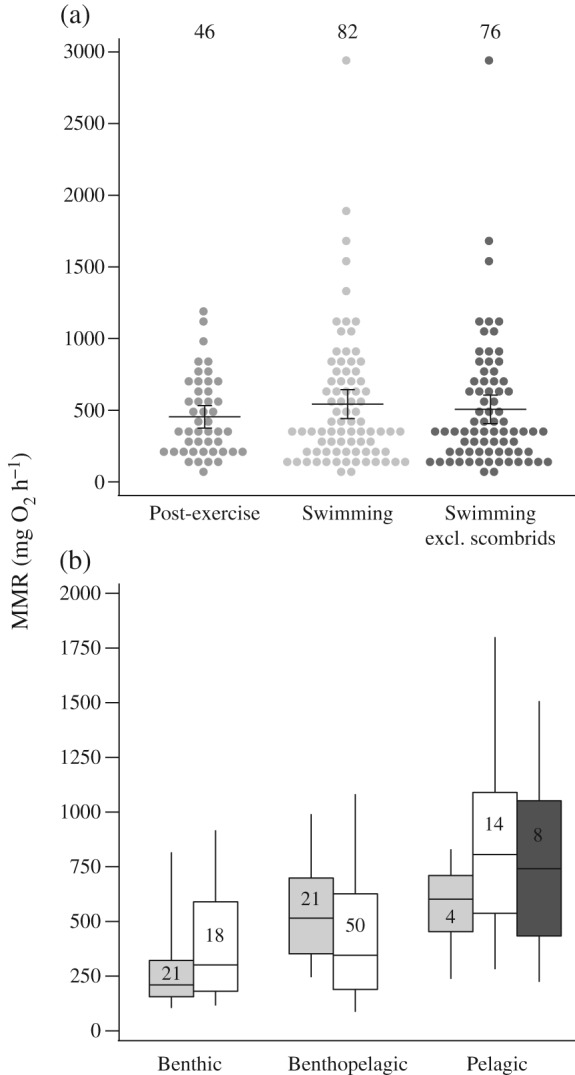
Comparisons of maximum metabolic rate (MMR) of fishes measured using either the swimming method or the post‐exercise method. Values are standardized to 1000 g mass and 20° C. (a) Comparison of MMR estimates obtained using the swimming and post‐exercise respirometry methods and a separate representation for the swimming respirometry method which excludes the high‐performance scombrid species. 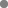, Post‐exercise, 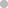, swimming and 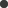, swimming excluding scombrids, values are MMR of species in 70 mg O_2_ h^−1^ bins; 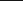, mean values; 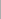, 95% c.i. (b) Box‐plot methodological comparisons (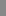, post‐exercise; 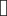, swimming; 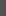, swimming, excluding scombrids) among fish species with different lifestyles (benthic, benthopelagic and pelagic). The middle line inside the boxes represents the median, while the lower and upper box boundaries represent the 25th and 75th percentiles, respectively. The lower and upper whiskers represent the 5th and 95th percentiles, respectively. Samples sizes are given as numerical values in both (a) and (b).

For the 14 species for which data for both the swimming and post‐exercise respirometry methods existed, there was no evidence of a difference in estimated MMR between the two methods (Fig. 2; standardized MMR for swimming respirometry method; mean ± s.e. = 567·71 ± 81·25 mg O_2_ h^−1^ and for post‐exercise respirometry method = 531·01 ± 59·56 mg O_2_ h^−1^; paired *t*‐test: *t* = 0·041, d.f. = 13, *P* > 0·05). The intercept of the regression line describing the relationship between estimates of MMR obtained by each method was not statistically significantly different from zero (95% c.i.: −307·14 to 215·12) and the regression slope was not significantly different from one (95% c.i.: 0·70 to 1·61; Fig. 2). The existing data therefore gives a nearly 1:1 relationship between MMR values obtained by each method for the same species, indicating that there is no systematic bias in the MMR estimates produced by the two methods. The direction of the difference between methods, however, varies among species and data for additional species are needed to distinguish whether any of these are true, taxa‐specific differences rather than noise. It is notable that six of the seven species that have a positive residual value in Fig. 2 are tropical fishes with a relatively high activity level, although much of these data come from a small number of studies (Roche *et al.*, 2013; Rummer *et al.*, 2016). In summary of the analysis, although a greater sample size could conceivably highlight an important difference, at present there is limited evidence from within‐ or between‐species analyses that the method employed for estimating MMR has a systematic effect on the values obtained.

**Figure 2:**
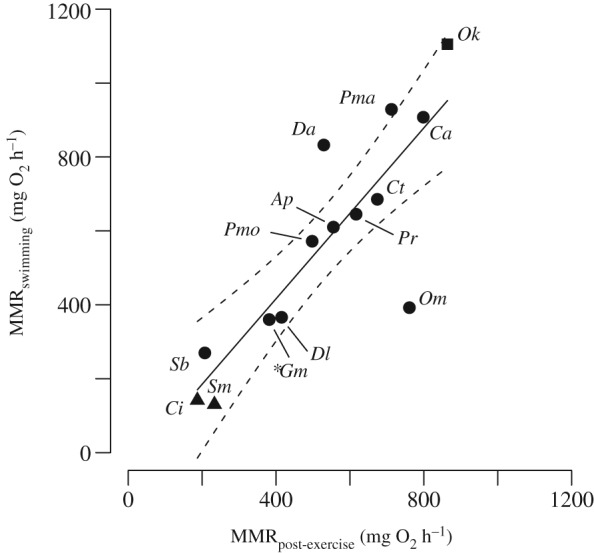
Scatterplot of fish species for which maximum metabolic rate (MMR) measures (standardized to 1000 g mass and 20° C) were available using both the swimming and post‐exercise respirometry method (ordinary least‐squares regression: y = −46·01 (±119·85 s.e.) + 1·16 (±0·21 s.e.) x; r ^2^ = 0·72, P < 0·001). 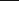, Regression line and 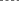, 95% c.i., as well as the abbreviated species identity of each data point: Ap, Acanthochromis polyacanthus; Ca, Chromis atripectoralis; Ci, Ctenopharyngodon idella; Ct, Caesio teres; Da, Dascyllus aruanus; Dl, Dicentrarchus labrax; Gm, Gadus morhua; Ok, Oncorhynchus kisutch; Om, Oncorhynchus mykiss; Pma, Pterocaesio marri; Pmo, Pomacentrus moluccensis; Pr, Poecilia reticulata; Sb, Scolopsis bilineata; Sm, Silurus meridionalis. 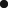, Benthopelagic species; 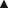, benthic species (Ctenopharyngodon idella and Silurus meridionalis); 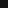, pelagic species (Oncorhynchus kisutch). N.B. Multiple MMR estimates were available for G. morhua from which a single mean value was calculated for each method. Literature references for these studies can be found in Table S1 (Supporting Information).

The overall similarity between protocols suggests that there may be other, more important factors than the choice of method to consider when measuring the MMR of fishes. Indeed, failure to adhere to best practices in the details of whichever respirometry method is chosen (Steffensen, 1989; Clark *et al.*, 2013; Norin & Clark, 2016; Rodgers *et al.*, 2016; Svendsen *et al.*, 2016) is likely to generate more variability in estimates of MMR than the broad choice of method. In the post‐exercise method, for example, oxygen uptake can decrease rapidly upon cessation of exercise (Norin & Clark, 2016) and so rapid transfer to the respirometry chamber is crucial, as any delay may cause the initial post‐exercise elevation in oxygen uptake to be missed. In some cases, it may even be necessary to measure ${\dot{M}}_{O_{2}}$ at sequential time intervals and then extrapolate to time zero post‐exercise to estimate MMR (Killen *et al.*, 2014). Ideally, researchers should measure and report the time taken from cessation of exercise to initiation of the first oxygen uptake recording when using the post‐exercise method. It should also be noted that in a few cases MMR has been reported to be delayed and not occur until a few hours after the cessation of exercise (Soofiani & Priede, 1985; Clark *et al.*, 2012). Although this appears atypical, measures of only a short duration after exercise may not be sufficient for accurately estimating MMR for some species.

There are undoubtedly species for which one method or the other is not logistically possible and so would not be effective at eliciting MMR. For example, obligate ram‐ventilating fishes are not suited for placement in a static‐water respirometer following a chase because they will not be able to ventilate their gills while remaining motionless. Similarly, many sedentary species will not swim against a current in a flume. The conclusion in the present study is that the available data offer little evidence that an obligation to use a particular method will bias the measures of MMR obtained. It is also worth considering, however, the possibility that neither the post‐exercise nor the swimming respirometry method gives true measures of the animal's actual MMR. Indeed, given the potential issues surrounding each method, largely stemming from the motivation of the animal to engage in exercise, it is possible that both methods may underestimate MMR. This issue is separate, however, from whether choice between currently available methods will bias MMR estimates. It is worth noting that alternative methods for estimating MMR are in use. For example, in a range of tropical coral‐reef fishes, MMR has been estimated using a cylindrical respirometry chamber within which a fish is required to swim against a vortex created by a rotating stir bar. This method has, however, been found to underestimate MMR when compared with the more established methods (Rummer *et al.*, 2016). There are also some fish species that appear to approach MMR during a post‐feeding increase in metabolism during the digestion and assimilation of nutrients (Soofiani & Hawkins, 1982; Fu *et al.*, 2008). Other species, however, reach a higher MMR when exhaustive exercise is combined with digestion (Dupont‐Prinet *et al.*, 2009; Jourdan‐Pineau *et al.*, 2010; Zhang *et al.*, 2012), so feeding‐induced increases in ${\dot{M}}_{O_{2}}$ are likely not reliable estimates of MMR in most fishes.

A source of discrepancy between measures obtained by the swimming and post‐exercise methods, where such differences exists, is probably the degree to which each method reflects a steady‐state of oxygen flux between the fish's tissues and the corresponding drop in oxygen content in the water surrounding the animal. During established critical swimming speed (*U*_crit_) protocols, for example, the animal swims at each stepwise speed until oxygen uptake from the water matches that consumed by the aerobic metabolic rate of the fish. During post‐exercise protocols, however, burst‐type swimming may rapidly deplete circulating blood and myoglobin oxygen content, causing a temporary disequilibrium between oxygen uptake from the water and actual mitochondrial oxygen usage to support metabolism (Farrell, 2016). This will be especially problematic when MMR measurements are attempted in respirometers that are too large for the animal of interest (Norin & Clark, 2016). A key consideration in this regard is how oxygen uptake post‐exercise is partitioned among various components of recovery, including actual swimming costs, metabolite processing and replenishment of myoglobin and arterial oxygen (Scarabello *et al.*, 1992; Farrell, 2016). Although the present analysis shows that there is generally little difference between the two methods, researchers must be cognizant of how oxygen uptake relates to aerobic metabolism during and after exercise and avoid issues that may cause estimates of MMR to be a methodological artefact.

In conclusion, for species amenable to either swimming respirometry or post exercise respirometry, it seems the benefits of the post‐exercise protocol may be warranted when considering the choice of method. In general, the available evidence suggests that both the swimming and post‐exercise respirometry methods will give similar estimates of MMR for most species and that researchers should focus on making their method of choice as accurate as possible using best practices.

Thanks to M. Ryan and J. Nati for assistance with data collection. Thanks also to N. Metcalfe, T. Clark and an anonymous reviewer for feedback on an earlier version of this manuscript. S.S.K was supported by NERC Advanced Fellowship NE/J019100/1 and European Research Council Starting Grant no. 640004. T.N. was supported by a DFF‐Individual Postdoctoral grant from the Danish Council for Independent Research (grant no. DFF‐4181‐00297).

## Supporting information


table S1. Summary of species by lifestyle group (B, benthic; BP, benthopelagic; P, pelagic) reviewed for the analysis of maximum metabolic rate (MMR) and its standardization to 1000 g mass at 20° C (MMR_20_).

fig. S1. Phylogenetic tree of the relationships between fish species included in the present study. Phylogenetically informed analyses were performed using the phylogenetic generalized least squares (PGLS) method (Grafen, 1989; Martins & Hansen, 1997; Garland & Ives, 2000) was employed via the ape package (Paradis et al., 2004) in R ( www.r‐project.org), applying a phylogeny generated from the comprehensive tree of life (Hinchliff et al., 2015) using the rotl package (Michonneau et al., 2016), which was then manually augmented. The branch lengths were estimated using Grafen's branch‐length transformation (Grafen, 1989) (branch lengths set to a length equal to the number of descendant tips minus one). The effect of method for estimating maximum metabolic rate (R_MMR_; swimming or post‐exercise method) was non‐significant whether the PGLS model included lifestyle (effect of method without lifestyle: t = −0·11, P > 0·05; effect of method with lifestyle included in model: t = 0·149, P > 0·05).
