# Supplementary material for: Do method and species lifestyle affect measures of maximum metabolic rate in fishes?
Source: J Fish Biol. 2016 Oct 25;90(3):1037–46. doi: 10.1111/jfb.13195 (PMC5347950; doi:10.1111/jfb.13195)
Supplement: Supplementary file 1 — table S1. Summary of species by lifestyle group (B, benthic; BP, benthopelagic; P, pelagic) reviewed for the analysis of maximum metabolic rate (MMR) and its standardization to 1000 g mass at 20° C (MMR20). [file JFB-90-1037-s001.docx]

TABLE SI. Summary of species by lifestyle group (B, benthic; BP, benthopelagic; P, pelagic) reviewed for the analysis of maximum metabolic rate (*R*_MM_) and its standardization to 1000 g mass at 20^o^ C (*R*_MM20_)

| Species | Order | Family | Lifestyle | *R*_MM_ | Mass  (g) | Temperature  (^o^C) | *R*_MM20_ | Method | Paired comparison? | Reference |
| --- | --- | --- | --- | --- | --- | --- | --- | --- | --- | --- |
| *Abbottina rivularis* | Cyrpiniformes | Cyprinidae | BP | 2.77 | 15.90 | 25.0 | 111.1 | Whilst swimming | No | Yan, G. J., He, X. K., Cao, Z. D., & Fu, S. J. (2013). An interspecific comparison between morphology and swimming performance in cyprinids. Journal of evolutionary biology, 26(8), 1802-1815. |
| *Acanthochromis polyacanthus* | Perciformes | Pomacentridae | BP | 17.69 | 16.88 | 28.5 | 555.5 | Post exhaustion | Yes | Rummer, J. L., Binning, S. A., Roche, D. G., & Johansen, J. L. (2016). Methods matter: considering locomotory mode and respirometry technique when estimating metabolic rates of fishes. Conservation Physiology 4, 1-13, doi: 10.1093/conphys/cow008. |
| *Acanthochromis polyacanthus* | Perciformes | Pomacentridae | BP | 19.42 | 16.88 | 28.5 | 609.9 | Whilst swimming | Yes | Rummer, J. L., Binning, S. A., Roche, D. G., & Johansen, J. L. (2016). Methods matter: considering locomotory mode and respirometry technique when estimating metabolic rates of fishes. Conservation Physiology 4, 1-13, doi: 10.1093/conphys/cow008. |
| *Acipenser fulvescens* | Acipenseriformes | Acipenseridae | B | 10.33 | 30.51 | 17.0 | 341.1 | Post exhaustion | No | Svendsen, J. C., Genz, J., Anderson,W. G., Stol, J. A.,Watkinson, D. A. & Enders, E. C. (2014). Evidence of circadian rhythm, oxygen regulation capacity, metabolic repeatability and positive correlations between forced and spontaneous maximal metabolic rates in lake sturgeon Acipenser fulvescens. PLoS One 9, e94693. doi: 10.1371/journal.pone.0094693 |
| *Albula vulpes* | Albuliformes | Albulidae | BP | 195.21 | 672.00 | 20.6 | 276.4 | Post exhaustion | No | Murchie, K. J., Cooke, S. J., Danylchuk, A. J. & Suski, C. D. (2011). Estimates of field activity and metabolic rates of bonefish (Albula vulpes) in coastal marine habitats using acoustic tri-axial accelerometer transmitters and intermittent-flow respirometry. Journal of Experimental Marine Biology and Ecology 396, 147–155. doi: 10.1016/j.jembe.2010.10.019 |
| *Ammodytes tobianus* | Perciformes | Ammodytidae | B | 1.59 | 3.30 | 10.0 | 641.6 | Post exhaustion | No | Behrens, J. W., & Steffensen, J. F. (2007). The effect of hypoxia on behavioural and physiological aspects of lesser sandeel, Ammodytes tobianus (Linnaeus, 1785). Marine Biology, 150(6), 1365-1377. |
| *Anarhichas lupus* | Perciformes | Anarhichadidae | B | 113.84 | 1000.00 | 10.0 | 194.6 | Post exhaustion | No | Liao, Y. Y., & Lucas, M. C. (2000). Growth, diet and metabolism of common wolf–fish in the North Sea, a fast–growing population. Journal of Fish Biology, 56(4), 810-825. |
| *Anguilla anguilla* | Anguilliformes | Anguillidae | B | 94.08 | 200.00 | 23.0 | 373.2 | Whilst swimming | No | McKenzie, D. J., Piccolella, M., Dalla Valle, A. Z., Taylor, E. W., Bolis, C. L., & Steffensen, J. F. (2003). Tolerance of chronic hypercapnia by the European eel Anguilla anguilla. Journal of experimental biology, 206(10), 1717-1726. |
| *Anguilla rostrata* | Anguilliformes | Anguillidae | B | 48.84 | 243.00 | 15.0 | 247.0 | Whilst swimming | No | Holmberg, B., & Saunders, R. L. (1979). The effects of pentachlorophenol on swimming performance and oxygen consumption in the American eel (Anguilla rostrata). Rapports et Proces Verbaux des Reunions. |
| *Argyrosomus japonicus* | Perciformes | Sciaenidae | BP | 124.10 | 340.00 | 22.0 | 312.7 | Whilst swimming | No | Fitzgibbon, Q. P., Strawbridge, A., & Seymour, R. S. (2007). Metabolic scope, swimming performance and the effects of hypoxia in the mulloway, Argyrosomus japonicus (Pisces: Sciaenidae). Aquaculture, 270(1), 358-368. |
| *Aristichthys nobilis* | Cyrpiniformes | Cyprinidae | BP | 1.44 | 6.19 | 25.0 | 142.8 | Whilst swimming | No | Yan, G. J., He, X. K., Cao, Z. D., & Fu, S. J. (2013). An interspecific comparison between morphology and swimming performance in cyprinids. Journal of evolutionary biology, 26(8), 1802-1815. |
| *Caesio teres* | Perciformes | Caesionidae | BP | 19.26 | 15.07 | 28.5 | 674.0 | Post exhaustion | Yes | Rummer, J. L., Binning, S. A., Roche, D. G., & Johansen, J. L. (2016). Methods matter: considering locomotory mode and respirometry technique when estimating metabolic rates of fishes. Conservation Physiology 4, 1-13, doi: 10.1093/conphys/cow008. |
| *Caesio teres* | Perciformes | Caesionidae | BP | 19.58 | 15.07 | 28.5 | 685.3 | Whilst swimming | Yes | Rummer, J. L., Binning, S. A., Roche, D. G., & Johansen, J. L. (2016). Methods matter: considering locomotory mode and respirometry technique when estimating metabolic rates of fishes. Conservation Physiology 4, 1-13, doi: 10.1093/conphys/cow008. |
| *Caranx caballus* | Perciformes | Carangidae | P | 110.68 | 190.00 | 27.2 | 368.2 | Whilst swimming | No | Dickson, K. A., Donley, J. M., Hansen, M. W. & Peters, J. A. (2012). Maximum sustainable speed, energetics and swimming kinematics of a tropical carangid fish, the green jack Caranx caballus. Journal of Fish Biology 80, 2494–2516. doi: 10.1111/j.1095-8649.2012.03302.x |
| *Carassius auratus* | Cyrpiniformes | Cyprinidae | BP | 1.61 | 6.22 | 25.0 | 158.8 | Whilst swimming | No | Yan, G. J., He, X. K., Cao, Z. D., & Fu, S. J. (2013). An interspecific comparison between morphology and swimming performance in cyprinids. Journal of evolutionary biology, 26(8), 1802-1815. |
| *Carassius carassius* | Cyrpiniformes | Cyprinidae | B | 1.78 | 6.06 | 25.0 | 179.9 | Post exhaustion | No | Fu, S. J., Zeng, L. Q., Li, X. M., Pang, X., Cao, Z. D., Peng, J. L., & Wang, Y. X. (2009). The behavioural, digestive and metabolic characteristics of fishes with different foraging strategies. Journal of Experimental Biology, 212(14), 2296-2302. |
| *Cheilinus fasciatus* | Perciformes | Labridae | BP | 22.49 | 34.20 | 27.7 | 375.3 | Whilst swimming | No | Fulton, C. J., Johansen, J. L.&Steffensen, J. F. (2013). Energetic extremes in aquatic locomotion by coral reef fishes. PLoS One 8, e54033. doi: 10.1371/journal.pone.0054033 |
| *Chromis atripectoralis* | Perciformes | Pomacentridae | BP | 12.61 | 8.10 | 28.5 | 798.9 | Post exhaustion | Yes | Rummer, J. L., Binning, S. A., Roche, D. G., & Johansen, J. L. (2016). Methods matter: considering locomotory mode and respirometry technique when estimating metabolic rates of fishes. Conservation Physiology 4, 1-13, doi: 10.1093/conphys/cow008. |
| *Chromis atripectoralis* | Perciformes | Pomacentridae | BP | 14.32 | 8.10 | 28.5 | 907.4 | Whilst swimming | Yes | Rummer, J. L., Binning, S. A., Roche, D. G., & Johansen, J. L. (2016). Methods matter: considering locomotory mode and respirometry technique when estimating metabolic rates of fishes. Conservation Physiology 4, 1-13, doi: 10.1093/conphys/cow008. |
| *Chromis ternatensis* | Perciformes | Pomacentridae | BP | 8.69 | 5.84 | 29.0 | 732.9 | Whilst swimming | No | Johansen, J. L. & Jones, G. P. (2011). Increasing ocean temperature reduces the metabolic performance and swimming ability of coral reef damselfishes. Global Change Biology 17, 2971–2979. doi: 10.1111/j.1365-2486.2011.02436.x |
| *Chromis veridis* | Perciformes | Pomacentridae | BP | 3.46 | 1.72 | 28.0 | 990.7 | Post exhaustion | No | Killen unpublished |
| *Coreius guichenoti* | Cyrpiniformes | Cyprinidae | BP | 37.04 | 67.30 | 25.0 | 373.9 | Whilst swimming | No | Tu, Z., Li, L., Yuan, X. I., Huang, Y., & Johnson, D. (2012). Aerobic swimming performance of juvenile Largemouth bronze gudgeon (Coreius guichenoti) in the Yangtze River. Journal of Experimental Zoology Part A: Ecological Genetics and Physiology, 317(5), 294-302. |
| *Ctenopharyngodon idella* | Cyrpiniformes | Cyprinidae | B | 2.25 | 7.42 | 25.0 | 187.1 | Post exhaustion | Yes | Fu, S. J., Zeng, L. Q., Li, X. M., Pang, X., Cao, Z. D., Peng, J. L., & Wang, Y. X. (2009). The behavioural, digestive and metabolic characteristics of fishes with different foraging strategies. Journal of Experimental Biology, 212(14), 2296-2302. |
| *Ctenopharyngodon idella* | Cyrpiniformes | Cyprinidae | B | 0.65 | 2.66 | 25.0 | 143.1 | Whilst swimming | Yes | Yan, G. J., He, X. K., Cao, Z. D., & Fu, S. J. (2013). An interspecific comparison between morphology and swimming performance in cyprinids. Journal of evolutionary biology, 26(8), 1802-1815. |
| *Cyclopterus lumpus* | Scorpaeniformes | Cycloperidae | BP | 130.18 | 782.00 | 11.0 | 266.8 | Post exhaustion | No | Killen, S. S., Costa, I., Brown, J. A., & Gamperl, A. K. (2007). Little left in the tank: metabolic scaling in marine teleosts and its implications for aerobic scope. Proceedings of the Royal Society of London B: Biological Sciences, 274(1608), 431-438. |
| *Cyprinus carpio* | Cyrpiniformes | Cyprinidae | BP | 1.10 | 5.15 | 25.0 | 130.1 | Whilst swimming | No | Yan, G. J., He, X. K., Cao, Z. D., & Fu, S. J. (2013). An interspecific comparison between morphology and swimming performance in cyprinids. Journal of evolutionary biology, 26(8), 1802-1815. |
| *Danio rerio* | Cyrpiniformes | Cyprinidae | BP | 0.61 | 0.50 | 24.0 | 707.0 | Post exhaustion | No | Lucas, M. C., & Priede, I. G. (1992). Utilization of metabolic scope in relation to feeding and activity by individual and grouped zebrafish, Brachydanio rerio (Hamilton‐Buchanan). Journal of fish biology, 41(2), 175-190. |
| *Dascyllus aruanus* | Perciformes | Pomacentridae | BP | 3.57 | 3.43 | 28.0 | 529.0 | Post exhaustion | Yes | Munday, P. L., Cheal, A. J., Dixson, D. L., Rummer, J. L., & Fabricius, K. E. (2014). Behavioural impairment in reef fishes caused by ocean acidification at CO2 seeps. Nature Climate Change, 4(6), 487-492. |
| *Dascyllus aruanus* | Perciformes | Pomacentridae | BP | 10.11 | 5.99 | 29.0 | 832.2 | Whilst swimming | Yes | Johansen, J. L. & Jones, G. P. (2011). Increasing ocean temperature reduces the metabolic performance and swimming ability of coral reef damselfishes. Global Change Biology 17, 2971–2979. doi: 10.1111/j.1365-2486.2011.02436.x |
| *Dascyllus reticulatus* | Perciformes | Pomacentridae | BP | 15.71 | 7.52 | 29.0 | 1040.4 | Whilst swimming | No | Johansen, J. L. & Jones, G. P. (2011). Increasing ocean temperature reduces the metabolic performance and swimming ability of coral reef damselfishes. Global Change Biology 17, 2971–2979. doi: 10.1111/j.1365-2486.2011.02436.x |
| *Dicentrarchus labrax* | Perciformes | Moronidae | BP | 20.05 | 42.00 | 20.0 | 415.3 | Post exhaustion | Yes | Killen_et_al_unpublished (same fish as in Killen, S. S., Marras, S., & McKenzie, D. J. (2011). Fuel, fasting, fear: routine metabolic rate and food deprivation exert synergistic effects on risk‐taking in individual juvenile European sea bass. Journal of animal ecology, 80(5), 1024-1033.) |
| *Dicentrarchus labrax* | Perciformes | Moronidae | BP | 42.48 | 147.00 | 14.0 | 366.4 | Whilst swimming | Yes | Claireaux, G., Couturier, C. & Groison, A.-L. (2006). Effect of temperature on maximum swimming speed and cost of transport in juvenile European sea bass (Dicentrarchus labrax). Journal of Experimental Biology 209, 3420–3428. doi: 10.1242/jeb.02346 |
| *Echeneis naucrates* | Perciformes | Echeneidae | BP | 35.41 | 160.75 | 24.0 | 164.0 | Whilst swimming | No | Steffensen, J. F., & Lomholt, J. P. (1983). Energetic cost of active branchial ventilation in the sharksucker, Echeneis naucrates. Journal of Experimental Biology, 103(1), 185-192. |
| *Epalzeorhynchos frenatus* | Cyrpiniformes | Cyprinidae | BP | 1.73 | 2.10 | 25.0 | 480.0 | Whilst swimming | No | Beamish, F. W. H. (1981). Swimming performance and metabolic rate of three tropical fishes in relation to temperature. Hydrobiologia, 83(2), 245-254. |
| *Esox lucius* | Esociformes | Esocidae | B | 67.52 | 666.00 | 15.0 | 130.2 | Post exhaustion | No | Armstrong, J. D., Priede, I. G., & Lucas, M. C. (1992). The link between respiratory capacity and changing metabolic demands during growth of northern pike, Esox lucius L. Journal of fish biology, 41, 65-65. |
| *Euthynnus affinis* | Perciformes | Scombridae | P | 1064.13 | 1000.00 | 24.0 | 858.8 | Whilst swimming | No | Sepulveda, C. H. U. G. E. Y., & Dickson, K. A. (2000). Maximum sustainable speeds and cost of swimming in juvenile kawakawa tuna (Euthynnus affinis) and chub mackerel (Scomber japonicus). Journal of Experimental Biology, 203(20), 3089-3101. |
| *Forsterygion lapillum* | Perciformes | Tripterygiidae | BP | 0.73 | 2.10 | 15.0 | 346.3 | Post exhaustion | No | Khan, J. R., & Herbert, N. A. (2012). The behavioural thermal preference of the common triplefin (Forsterygion lapillum) tracks aerobic scope optima at the upper thermal limit of its distribution. Journal of Thermal Biology, 37(2), 118-124. |
| *Gadus macrocephalus* | Gadiformes | Gadidae | B | 193.73 | 1000.00 | 11.0 | 313.8 | Post exhaustion | No | Hanna, S. K., Haukenes, A. H., Foy, R. J., & Buck, C. L. (2008). Temperature effects on metabolic rate, swimming performance and condition of Pacific cod Gadus macrocephalus Tilesius. Journal of Fish Biology, 72(4), 1068-1078. |
| *Gadus morhua* | Gadiformes | Gadidae | BP | 58.97 | 150.00 | 15.0 | 472.9 | Post exhaustion | Yes | Soofiani, N. M., & Priede, I. G. (1985). Aerobic metabolic scope and swimming performance in juvenile cod, Gadus morhua L. Journal of Fish Biology, 26(2), 127-138. |
| *Gadus morhua* | Gadiformes | Gadidae | BP | 207.13 | 1000.00 | 11.0 | 335.6 | Whilst swimming | Yes | Sylvestre, E.-L., Lapointe, D., Dutil, J.-D. & Guderley, H. (2007). Thermal sensitivity of metabolic rates and swimming performance in two latitudinal separated populations of cod, Gadus morhua L. Journal of Comparative Physiology B 177, 447–460. doi: 10.1007/s00360-007-0143-x |
| *Gadus ogac* | Gadiformes | Gadidae | B | 28.46 | 180.00 | 4.0 | 345.7 | Post exhaustion | No | Bushnell, P. G., Steffensen, J. F., Schurmann, H., & Jones, D. R. (1994). Exercise metabolism in two species of cod in arctic waters. Polar Biology, 14(1), 43-48. |
| *Gasterosteus aculeautus* | Gasterosteiformes | Gasterosteidae | BP | 5.13 | 3.33 | 15.0 | 1568.7 | Whilst swimming | No | Tudorache, C., Blust, R. & De Boeck, G. (2007). Swimming capacity and energetics of migrating and non-migrating morphs of three-spined stickleback Gasterosteus aculeatus L. and their ecological implications. Journal of Fish Biology 71, 1448–1456. doi: 10.1111/j.1095-8649.2007.01612.x |
| *Hypophthalmichthys sp* | Cyrpiniformes | Cyprinidae | BP | 1.16 | 6.09 | 25.0 | 116.6 | Whilst swimming | No | Yan, G. J., He, X. K., Cao, Z. D., & Fu, S. J. (2013). An interspecific comparison between morphology and swimming performance in cyprinids. Journal of evolutionary biology, 26(8), 1802-1815. |
| *Ictalurus nebulosus* | Siluriformes | Ictaluridae | B | 79.98 | 200.00 | 20.0 | 372.6 | Whilst swimming | No | Saunders, R. L. (1962). THE IRRIGATION OF THE GILLS IN FISHES: II. EFFICIENCY OF OXYGEN UPTAKE IN RELATION TO RESPIRATORY FLOW ACTIVITY AND CONCENTRATIONS OF OXYGEN AND CARBON DIOXIDE. Canadian Journal of Zoology, 40(5), 817-862. |
| *Katsuwonus pelamis* | Perciformes | Scombridae | P | 3740.00 | 1700.00 | 23.0 | 1917.3 | Whilst swimming | No | Dewar, H., & Graham, J. (1994). Studies of tropical tuna swimming performance in a large water tunnel-energetics. The Journal of experimental biology, 192(1), 13-31. |
| *Kuhlia sandvicensis* | Perciformes | Kuhliidae | P | 19.62 | 30.00 | 23.0 | 477.4 | Whilst swimming | No | Muir, B. S., & Niimi, A. J. (1972). Oxygen consumption of the euryhaline fish aholehole (Kuhlia sandvicensis) with reference to salinity, swimming, and food consumption. Journal of the Fisheries Board of Canada, 29(1), 67-77. |
| *Lates calcarifer* | Perciformes | Latidae | B | 13.35 | 25.40 | 29.0 | 276.2 | Post exhaustion | No | Norin, T., Malte, H. & Clark, T. D. (2014). Aerobic scope does not predict the performance of am tropical eurythermal fish at elevated temperatures. Journal of Experimental Biology 217, 244–251. doi: 10.1242/jeb.089755 |
| *Leiostomus xanthrus* | Perciformes | Sciaenidae | B | 154.26 | 121.00 | 25.0 | 888.8 | Whilst swimming | No | Horodysky, A. Z., Brill, R. W., Bushnell, P. G., Musick, J. A., & Latour, R. J. (2011). Comparative metabolic rates of common western North Atlantic Ocean sciaenid fishes. Journal of fish biology, 79(1), 235-255. |
| *Lepomis gibbosus* | Perciformes | Centrarchidae | BP | 18.33 | 44.92 | 20.0 | 356.0 | Whilst swimming | No | Brett, J. R., & Sutherland, D. B. (1965). Respiratory metabolism of pumpkinseed (Lepomis gibbosus) in relation to swimming speed. Journal of the Fisheries Board of Canada, 22(2), 405-409. |
| *Limanda limanda* | Pleuronectiformes | Pleuronectidae | B | 67.83 | 395.80 | 15.0 | 215.1 | Whilst swimming | No | Duthie, G. G. (1982). The respiratory metabolism of temperature-adapted flatfish at rest and during swimming activity and the use of anaerobic metabolism at moderate swimming speeds. Journal of Experimental Biology, 97(1), 359-373. |
| *Liza aurata* | Mugiliformes | Mugilidae | P | 9.04 | 13.44 | 20.0 | 556.7 | Post exhaustion | No | Killen, S. S., Marras, S., Steffensen, J. F., & McKenzie, D. J. (2011). Aerobic capacity influences the spatial position of individuals within fish schools. Proceedings of the Royal Society of London B: Biological Sciences, rspb20111006. |
| *Maccullochella peelii peelii* | Perciformes | Percichthyidae | B | 219.29 | 1810.00 | 15.0 | 162.6 | Whilst swimming | No | Clark, T. D., Ryan, T., Ingram, B. A., Woakes, A. J., Butler, P. J. & Frappell, P. B. (2005). Factorial aerobic scope is independent of temperature and primarily modulated by heart rate in exercising Murray cod (Maccullochella peelii peelii). Physiological and Biochemical Zoology 78, 347–355. |
| *Macrozoraces americanus* | Perciformes | Zoarcidae | B | 250.09 | 1000.00 | 8.0 | 475.8 | Post exhaustion | No | Killen, S. S., Costa, I., Brown, J. A., & Gamperl, A. K. (2007). Little left in the tank: metabolic scaling in marine teleosts and its implications for aerobic scope. Proceedings of the Royal Society of London B: Biological Sciences, 274(1608), 431-438. |
| *Melanogrammus aeglefinus* | Gadiformes | Gadidae | BP | 44.33 | 155.92 | 10.0 | 447.9 | Whilst swimming | No | Tytler, P. (1969). Relationship between oxygen consumption and swimming speed in the haddock, Melanogrammus aeglefinus. Nature, 221, 274-275. |
| *Melanotaenia fluviatilis* | Atheriniformes | Melanotaeniidae | BP | 1.89 | 3.00 | 25.0 | 373.6 | Whilst swimming | No | Stoffels, R. J. (2015). Physiological Trade-Offs Along a Fast-Slow Lifestyle Continuum in Fishes: What Do They Tell Us about Resistance and Resilience to Hypoxia?. PloS one, 10(6), e0130303. |
| *Merlangius merlangus* | Gadiformes | Gadidae | BP | 47.70 | 297.00 | 10.0 | 260.3 | Whilst swimming | No | Steinhausen, M. F., Steffensen, J. F., & Andersen, N. G. (2005). Tail beat frequency as a predictor of swimming speed and oxygen consumption of saithe (Pollachius virens) and whiting (Merlangius merlangus) during forced swimming. Marine Biology, 148(1), 197-204. |
| *Micropogonias undulatus* | Perciformes | Sciaenidae | B | 238.16 | 274.00 | 25.0 | 628.1 | Whilst swimming | No | Horodysky, A. Z., Brill, R. W., Bushnell, P. G., Musick, J. A., & Latour, R. J. (2011). Comparative metabolic rates of common western North Atlantic Ocean sciaenid fishes. Journal of fish biology, 79(1), 235-255. |
| *Micropterus dolomieu* | Perciformes | Centrarchidae | BP | 201.22 | 516.00 | 20.4 | 370.8 | Post exhaustion | No | Peake, S. J. & Farrell, A. P. (2004). Locomotory behaviour and post-exercise physiology in relation to swimming speed, gait transition and metabolism in free-swimming smallmouth bass (Micropterus dolomieu). Journal of Experimental Biology 207, 1563–1575. doi: 10.1242/jeb.00927 |
| *Micropterus salmoides* | Perciformes | Centrarchidae | BP | 260.00 | 1000.00 | 15.0 | 339.9 | Whilst swimming | No | Beamish, F. W. H. (1970). Oxygen consumption of largemouth bass, Micropterus salmoides, in relation to swimming speed and temperature. Canadian Journal of Zoology, 48(6), 1221-1228. |
| *Microstomus kitt* | Pleuronectiformes | Pleuronectidae | B | 36.43 | 221.30 | 15.0 | 201.4 | Whilst swimming | No | Duthie, G. G. (1982). The respiratory metabolism of temperature-adapted flatfish at rest and during swimming activity and the use of anaerobic metabolism at moderate swimming speeds. Journal of Experimental Biology, 97(1), 359-373. |
| *Mogurnda adspersa* | Perciformes | Eleotridae | BP | 1.18 | 3.00 | 25.0 | 233.9 | Whilst swimming | No | Stoffels, R. J. (2015). Physiological Trade-Offs Along a Fast-Slow Lifestyle Continuum in Fishes: What Do They Tell Us about Resistance and Resilience to Hypoxia?. PloS one, 10(6), e0130303. |
| *Morone americana* | Perciformes | Moronidae | B | 27.04 | 81.72 | 15.0 | 387.5 | Whilst swimming | No | Neumann, D. A., O'Connor, J. M., & Sherk, J. A. (1981). Oxygen consumption of white perch (Morone americana), striped bass (M. saxatius) and spot (Leiostomus xanthurus). Comparative Biochemistry and Physiology Part A: Physiology, 69(3), 467-478. |
| *Morone saxatilis* | Perciformes | Moronidae | B | 119.86 | 216.25 | 15.0 | 677.5 | Whilst swimming | No | Neumann, D. A., O'Connor, J. M., & Sherk, J. A. (1981). Oxygen consumption of white perch (Morone americana), striped bass (M. saxatius) and spot (Leiostomus xanthurus). Comparative Biochemistry and Physiology Part A: Physiology, 69(3), 467-478. |
| *Moxostoma anisurum* | Cypriniformes | Catostomidae | B | 74.26 | 728.00 | 13.4 | 143.3 | Post exhaustion | No | Hatry, C., Thiem, J. D., Binder, T. R., Hatin, D., Dumont, P., Stamplecoskie, K. M., ... & Cooke, S. J. (2014). Comparative Physiology and Relative Swimming Performance of Three Redhorse (Moxostoma spp.) Species: Associations with Fishway Passage Success*. Physiological and Biochemical Zoology, 87(1), 148-159. |
| *Moxostoma carinatum* | Cypriniformes | Catostomidae | B | 281.48 | 2182.00 | 15.8 | 167.2 | Post exhaustion | No | Hatry, C., Thiem, J. D., Binder, T. R., Hatin, D., Dumont, P., Stamplecoskie, K. M., ... & Cooke, S. J. (2014). Comparative Physiology and Relative Swimming Performance of Three Redhorse (Moxostoma spp.) Species: Associations with Fishway Passage Success*. Physiological and Biochemical Zoology, 87(1), 148-159. |
| *Moxostoma macrolepidotum* | Cypriniformes | Catostomidae | B | 57.76 | 380.00 | 13.7 | 204.2 | Post exhaustion | No | Hatry, C., Thiem, J. D., Binder, T. R., Hatin, D., Dumont, P., Stamplecoskie, K. M., ... & Cooke, S. J. (2014). Comparative Physiology and Relative Swimming Performance of Three Redhorse (Moxostoma spp.) Species: Associations with Fishway Passage Success*. Physiological and Biochemical Zoology, 87(1), 148-159. |
| *Mylopharyngodon piceus* | Cyrpiniformes | Cyprinidae | B | 1.96 | 7.05 | 25.0 | 170.7 | Whilst swimming | No | Yan, G. J., He, X. K., Cao, Z. D., & Fu, S. J. (2013). An interspecific comparison between morphology and swimming performance in cyprinids. Journal of evolutionary biology, 26(8), 1802-1815. |
| *Myoxocephalus scorpius* | Scorpaeniformes | Cottidae | B | 23.19 | 169.00 | 3.0 | 315.8 | Post exhaustion | No | Killen, S. S., Costa, I., Brown, J. A., & Gamperl, A. K. (2007). Little left in the tank: metabolic scaling in marine teleosts and its implications for aerobic scope. Proceedings of the Royal Society of London B: Biological Sciences, 274(1608), 431-438. |
| *Neopomacentrus azysron* | Perciformes | Pomacentridae | BP | 299.64 | 227.20 | 29.0 | 762.8 | Whilst swimming | No | Johansen, J. L. & Jones, G. P. (2011). Increasing ocean temperature reduces the metabolic performance and swimming ability of coral reef damselfishes. Global Change Biology 17, 2971–2979. doi: 10.1111/j.1365-2486.2011.02436.x |
| *Neopomacentrus benkieri* | Perciformes | Pomacentridae | BP | 9.90 | 5.94 | 29.0 | 821.5 | Whilst swimming | No | Johansen, J. L. & Jones, G. P. (2011). Increasing ocean temperature reduces the metabolic performance and swimming ability of coral reef damselfishes. Global Change Biology 17, 2971–2979. doi: 10.1111/j.1365-2486.2011.02436.x |
| *Neopomacentrus cyanomos* | Perciformes | Pomacentridae | BP | 7.08 | 5.68 | 29.0 | 613.4 | Whilst swimming | No | Johansen, J. L. & Jones, G. P. (2011). Increasing ocean temperature reduces the metabolic performance and swimming ability of coral reef damselfishes. Global Change Biology 17, 2971–2979. doi: 10.1111/j.1365-2486.2011.02436.x |
| *Notothenia neglecta* | Perciformes | Nototheniidae | B | 78.30 | 190.00 | 0.5 | 1089.7 | Whilst swimming | No | Johnston, I. A., Clarke, A., & Ward, P. (1991). Temperature and metabolic rate in sedentary fish from the Antarctic, North Sea and Indo-West Pacific Ocean. Marine Biology, 109(2), 191-195. |
| *Oncorhynchus gorbuscha* | Salmoniformes | Salmonidae | P | 2039.04 | 1600.00 | 15.0 | 1700.9 | Whilst swimming | No | Clark, T. D., Jeffries, K. M., Hinch, S. G., & Farrell, A. P. (2011). Exceptional aerobic scope and cardiovascular performance of pink salmon (Oncorhynchus gorbuscha) may underlie resilience in a warming climate. The Journal of Experimental Biology, 214(18), 3074-3081. |
| *Oncorhynchus kisutch* | Salmoniformes | Salmonidae | P | 938.36 | 2260.00 | 7.0 | 863.9 | Post exhaustion | Yes | Clark, T. D., Donaldson M. R., Pieperhoff, S., Drenner S. M., Lotto, A., Cooke, S. J., Hinch, S. G., Patterson, D. A. & Farrell, A. P. (2012). Physiological benefits of being small in a changing world: responses of coho salmon (Oncorhynchus kisutch) to an acute thermal challenge and a simulated capture event, PLoS One 7(6): e39079. |
| *Oncorhynchus kisutch* | Salmoniformes | Salmonidae | P | 1281.33 | 2300.00 | 7.9 | 1105.4 | Whilst swimming | Yes | Lee, C. G., Farrell, A. P., Lotto, A., MacNutt, M. J., Hinch, S. G., & Healey, M. C. (2003). The effect of temperature on swimming performance and oxygen consumption in adult sockeye (Oncorhynchus nerka) and coho (O. kisutch) salmon stocks. Journal of Experimental Biology, 206(18), 3239-3251. |
| *Oncorhynchus mykiss* | Salmoniformes | Salmonidae | BP | 22.70 | 33.60 | 15.0 | 761.0 | Post exhaustion | Yes | Chen, Z., Snow, M., Lawrence, C. S., Church, A. R., Narum, S. R., Devlin, R. H., Farrell, A. P. (2015) Selection for upper thermal tolerance in rainbow trout (*Oncorhynchus mykiss* Walbaum). Journal of Experimental Biology 218, 803-812. |
| *Oncorhynchus mykiss* | Salmoniformes | Salmonidae | BP | 351.72 | 1117.00 | 16.0 | 392.1 | Whilst swimming | Yes | Claireaux, G., McKenzie, D. J., Genge, A. G., Chatelier, A., Aubin, J., & Farrell, A. P. (2005). Linking swimming performance, cardiac pumping ability and cardiac anatomy in rainbow trout. Journal of Experimental Biology 208, 1775-1784. |
| *Oncorhynchus nerka* | Salmoniformes | Salmonidae | P | 1995.84 | 2640.00 | 15.0 | 1031.4 | Whilst swimming | No | Lee, C. G., Farrell, A. P., Lotto, A., MacNutt, M. J., Hinch, S. G., & Healey, M. C. (2003). The effect of temperature on swimming performance and oxygen consumption in adult sockeye (Oncorhynchus nerka) and coho (O. kisutch) salmon stocks. Journal of Experimental Biology, 206(18), 3239-3251. |
| *Onychostoma sp* | Cyrpiniformes | Cyprinidae | BP | 1.06 | 2.74 | 25.0 | 228.4 | Whilst swimming | No | Yan, G. J., He, X. K., Cao, Z. D., & Fu, S. J. (2013). An interspecific comparison between morphology and swimming performance in cyprinids. Journal of evolutionary biology, 26(8), 1802-1815. |
| *Opsariichthys bidens* | Cyrpiniformes | Cyprinidae | BP | 3.81 | 11.70 | 25.0 | 204.9 | Whilst swimming | No | Yan, G. J., He, X. K., Cao, Z. D., & Fu, S. J. (2013). An interspecific comparison between morphology and swimming performance in cyprinids. Journal of evolutionary biology, 26(8), 1802-1815. |
| *Oreochromis niloticus* | Perciformes | Cichlididae | BP | 316.23 | 1000.00 | 15.0 | 413.4 | Whilst swimming | No | Farmer, G. J., & Beamish, F. W. H. (1969). Oxygen consumption of Tilapia nilotica in relation to swimming speed and salinity. Journal of the Fisheries Board of Canada, 26(11), 2807-2821. |
| *Pagothenia borchgrevinki* | Perciformes | Nototheniidae | BP | 91.73 | 1000.00 | 0.0 | 268.0 | Whilst swimming | No | Forster, M. E., Franklin, C. E., Taylor, H. H., & Davison, W. (1987). The aerobic scope of an Antarctic fish, Pagothenia borchgrevinki and its significance for metabolic cold adaptation. Polar Biology, 8(2), 155-159. |
| *Pagrus auratus* | Perciformes | Sparidae | BP | 61.83 | 179.00 | 18.0 | 356.5 | Post exhaustion | No | Cook, D. G., Wells, R. M., & Herbert, N. A. (2011). Anaemia adjusts the aerobic physiology of snapper (Pagrus auratus) and modulates hypoxia avoidance behaviour during oxygen choice presentations. The Journal of experimental biology, 214(17), 2927-2934. |
| *Pelteobagrus vachelli* | Siluriformes | Bagridae | B | 1.00 | 6.03 | 25.0 | 101.8 | Post exhaustion | No | Fu, S. J., Zeng, L. Q., Li, X. M., Pang, X., Cao, Z. D., Peng, J. L., & Wang, Y. X. (2009). The behavioural, digestive and metabolic characteristics of fishes with different foraging strategies. Journal of Experimental Biology, 212(14), 2296-2302. |
| *Perca fluviatilis* | Perciformes | Percidae | B | 63.78 | 223.00 | 23.0 | 228.0 | Post exhaustion | No | Brijs, J., Jutfelt, F., Clark, T. D., Gräns, A., Ekström, A., & Sandblom, E. (2015). Experimental manipulations of tissue oxygen supply do not affect warming tolerance of European perch. The Journal of Experimental Biology, jeb-121889. |
| *Phoxinus phoxinus* | Cypriniformes | Cyprinidae | B | 0.86 | 1.03 | 14.5 | 821.3 | Post exhaustion | No | Killen, S. S., Nati, J. J., & Suski, C. D. (2015, August). Vulnerability of individual fish to capture by trawling is influenced by capacity for anaerobic metabolism. In Proc. R. Soc. B (Vol. 282, No. 1813, p. 20150603). The Royal Society. |
| *Platichthys flesus* | Pleuronectiformes | Pleuronectidae | B | 84.92 | 395.00 | 15.0 | 269.8 | Whilst swimming | No | Duthie, G. G. (1982). The respiratory metabolism of temperature-adapted flatfish at rest and during swimming activity and the use of anaerobic metabolism at moderate swimming speeds. Journal of Experimental Biology, 97(1), 359-373. |
| *Pleuronectes platessa* | Pleuronectiformes | Pleuronectidae | B | 50.54 | 280.80 | 15.0 | 222.6 | Whilst swimming | No | Priede, I. G., & Holliday, F. G. T. (1980). The use of a new tilting tunnel respirometer to investigate some aspects of metabolism and swimming activity of the plaice (Pleuronectes platessa L.). J. exp. Biol, 85, 295-309. |
| *Poecilia reticulata* | Cyrpinodontiformes | Poeciliidae | BP | 0.47 | 0.41 | 25.0 | 617.0 | Post exhaustion | Yes | Killen unpublished |
| *Poecilia reticulata* | Cyrpinodontiformes | Poeciliidae | BP | 0.38 | 0.30 | 26.0 | 644.9 | Whilst swimming | Yes | Svendsen, J. C., Banet, A. I., Christensen, R. H. B., Steffensen, J. F. & Aarestrup, K. (2013). Effects of intraspecific variation in reproductive traits, pectoral fin use and burst swimming on metabolic rates and swimming performance in the Trinidadian guppy (Poecilia reticulata). Journal of Experimental Biology 216, 3564–3574. doi: 10.1242/jeb.083089 |
| *Pollachius virens* | Gadiformes | Gadidae | B | 152.73 | 485.00 | 10.0 | 521.4 | Whilst swimming | No | Steinhausen, M. F., Steffensen, J. F., & Andersen, N. G. (2005). Tail beat frequency as a predictor of swimming speed and oxygen consumption of saithe (Pollachius virens) and whiting (Merlangius merlangus) during forced swimming. Marine Biology, 148(1), 197-204. |
| *Pomacentrus amboinensis* | Perciformes | Pomacentridae | BP | 0.20 | 0.07 | 29.0 | 1155.9 | Post exhaustion | No | Killen, S. S., Mitchell, M. D., Rummer, J. L., Chivers, D. P., Ferrari, M. C., Meekan, M. G., & McCormick, M. I. (2014). Aerobic scope predicts dominance during early life in a tropical damselfish. Functional Ecology, 28(6), 1367-1376. |
| *Pomacentrus coelestis* | Perciformes | Pomacentridae | BP | 12.06 | 5.32 | 29.0 | 1111.6 | Whilst swimming | No | Johansen, J. L. & Jones, G. P. (2011). Increasing ocean temperature reduces the metabolic performance and swimming ability of coral reef damselfishes. Global Change Biology 17, 2971–2979. doi: 10.1111/j.1365-2486.2011.02436.x |
| *Pomacentrus lipedogenys* | Perciformes | Pomacentridae | BP | 4.52 | 6.16 | 29.0 | 362.3 | Whilst swimming | No | Johansen, J. L. & Jones, G. P. (2011). Increasing ocean temperature reduces the metabolic performance and swimming ability of coral reef damselfishes. Global Change Biology 17, 2971–2979. doi: 10.1111/j.1365-2486.2011.02436.x |
| *Pomacentrus moluccensis* | Perciformes | Pomacentridae | BP | 4.74 | 4.91 | 28.0 | 498.1 | Post exhaustion | Yes | Munday, P. L., Cheal, A. J., Dixson, D. L., Rummer, J. L., & Fabricius, K. E. (2014). Behavioural impairment in reef fishes caused by ocean acidification at CO2 seeps. Nature Climate Change, 4(6), 487-492. |
| *Pomacentrus moluccensis* | Perciformes | Pomacentridae | BP | 6.03 | 5.16 | 29.0 | 572.2 | Whilst swimming | Yes | Johansen, J. L. & Jones, G. P. (2011). Increasing ocean temperature reduces the metabolic performance and swimming ability of coral reef damselfishes. Global Change Biology 17, 2971–2979. doi: 10.1111/j.1365-2486.2011.02436.x |
| *Pomatomus saltatrix* | Perciformes | Pomatomidae | P | 148.47 | 221.10 | 15.0 | 821.6 | Whilst swimming | No | Freadman, M. A. (1981). Swimming energetics of striped bass (Morone saxatilis) and bluefish (Pomatomus saltatrix): hydrodynamic correlates of locomotion and gill ventilation. Journal of Experimental Biology, 90(1), 253-265. |
| *Procypris rabaudi* | Cyrpiniformes | Cyprinidae | BP | 0.62 | 3.64 | 25.0 | 102.6 | Whilst swimming | No | Yan, G. J., He, X. K., Cao, Z. D., & Fu, S. J. (2013). An interspecific comparison between morphology and swimming performance in cyprinids. Journal of evolutionary biology, 26(8), 1802-1815. |
| *Pseudorasbora parva* | Cyrpiniformes | Cyprinidae | BP | 0.91 | 3.29 | 25.0 | 164.3 | Whilst swimming | No | Yan, G. J., He, X. K., Cao, Z. D., & Fu, S. J. (2013). An interspecific comparison between morphology and swimming performance in cyprinids. Journal of evolutionary biology, 26(8), 1802-1815. |
| *Pterocaesio marri* | Perciformes | Caesionidae | BP | 13.61 | 9.89 | 28.5 | 712.6 | Post exhaustion | Yes | Rummer, J. L., Binning, S. A., Roche, D. G., & Johansen, J. L. (2016). Methods matter: considering locomotory mode and respirometry technique when estimating metabolic rates of fishes. Conservation Physiology 4, 1-13, doi: 10.1093/conphys/cow008. |
| *Pterocaesio marri* | Perciformes | Caesionidae | BP | 17.75 | 9.89 | 28.5 | 929.0 | Whilst swimming | Yes | Rummer, J. L., Binning, S. A., Roche, D. G., & Johansen, J. L. (2016). Methods matter: considering locomotory mode and respirometry technique when estimating metabolic rates of fishes. Conservation Physiology 4, 1-13, doi: 10.1093/conphys/cow008. |
| *Rhinecanthus aculeatus* | Tetraodontiformes | Balistidae | BP | 120.53 | 136.00 | 26.5 | 573.1 | Whilst swimming | No | Korsmeyer, K. E., Steffensen, J. F. & Herskin, J. (2002). Energetics of median and paired fin swimming, body and caudal fin swimming, and gait transition in parrotfish (Scarus schlegeli) and triggerfish (Rhinecanthus aculeatus). Journal of Experimental Biology 205, 1253–1263. |
| *Rhinichthys osculus* | Cyrpiniformes | Cyprinidae | B | 1.37 | 2.44 | 12.0 | 659.6 | Whilst swimming | No | Rajagopal, P. K., & Kramer, R. H. (1974). Respiratory metabolism of Utah chub, Gila atraria (Girard) and speckled dace, Rhinichthys osculus (Girard). Journal of Fish Biology, 6(2), 215-222. |
| *Rhinomugil corsula* | Mugiliformes | Mugilidae | P | 15.84 | 44.60 | 30.0 | 181.2 | Whilst swimming | No | Sukumaran, N., & Kutty, M. N. (1979). Oxygen consumption and nitrogen excretion in mullet, Rhinomugil corsula (Hamilton), with special reference to swimming speed. Proceedings: Animal Sciences, 88(5), 345-351. |
| *Rhodeus ocellatus* | Cyrpiniformes | Cyprinidae | BP | 1.09 | 3.93 | 25.0 | 166.8 | Whilst swimming | No | Yan, G. J., He, X. K., Cao, Z. D., & Fu, S. J. (2013). An interspecific comparison between morphology and swimming performance in cyprinids. Journal of evolutionary biology, 26(8), 1802-1815. |
| *Rhodeus sinensis* | Cyrpiniformes | Cyprinidae | BP | 1.99 | 8.94 | 25.0 | 138.7 | Whilst swimming | No | Yan, G. J., He, X. K., Cao, Z. D., & Fu, S. J. (2013). An interspecific comparison between morphology and swimming performance in cyprinids. Journal of evolutionary biology, 26(8), 1802-1815. |
| *Salmo salar* | Salmoniformes | Salmonidae | BP | 5.53 | 11.75 | 12.0 | 595.0 | Post exhaustion | No | Anttila, K., Jørgensen, S. M., Casselman, M. T., Timmerhaus, G., Farrell, A. P., & Takle, H. (2014). Association between swimming performance, cardiorespiratory morphometry, and thermal tolerance in Atlantic salmon (Salmo salar L.). Frontiers in Marine Science, 1, 76. |
| *Salmo trutta* | Salmoniformes | Salmonidae | P | 20.89 | 35.20 | 15.0 | 669.9 | Post exhaustion | No | Norin, T., & Malte, H. (2011). Repeatability of standard metabolic rate, active metabolic rate and aerobic scope in young brown trout during a period of moderate food availability. The Journal of experimental biology, 214(10), 1668-1675. |
| *Salvelinus alpinus* | Salmoniformes | Salmonidae | BP | 133.35 | 210.00 | 15.0 | 775.2 | Whilst swimming | No | Beamish, F. W.H. 1980. Swimming performance and oxygen consumption within the genus Salvelinus. In Charrs: salmonid fishes of the genus Salvelinus. Edited by E.K. Balon. Dr. W. Junk Publishers, The Hague, The Netherlands. gp. 739-748. |
| *Salvelinus fontinalis* | Salmoniformes | Salmonidae | B | 12.08 | 11.30 | 15.0 | 1147.7 | Post exhaustion | No | Powell, M. D., Speare, D. J., Daley, J., & Lovy, J. (2005). Differences in metabolic response to Loma salmonae infection in juvenile rainbow trout Oncorhynchus mykiss and brook trout Salvelinus fontinalis. Diseases of aquatic organisms, 67(3), 233. |
| *Salvelinus namaycush* | Salmoniformes | Salmonidae | BP | 42.00 | 116.05 | 15.0 | 430.5 | Post exhaustion | No | Kelly, N. I., Burness, G., McDermid, J. L., & Wilson, C. C. (2014). Ice age fish in a warming world: minimal variation in thermal acclimation capacity among lake trout (Salvelinus namaycush) populations. Conservation Physiology, 2(1), cou025. |
| *Sander lucioperca* | Perciformes | Percidae | P | 166.90 | 1000.00 | 16.0 | 206.8 | Post exhaustion | No | Frisk, M., Skov, P. V., & Steffensen, J. F. (2012). Thermal optimum for pikeperch (Sander lucioperca) and the use of ventilation frequency as a predictor of metabolic rate. Aquaculture, 324, 151-157. |
| *Scarus schlegeli* | Perciformes | Scaridae | BP | 117.81 | 243.00 | 26.5 | 321.6 | Whilst swimming | No | Korsmeyer, K. E., Steffensen, J. F., & Herskin, J. (2002). Energetics of median and paired fin swimming, body and caudal fin swimming, and gait transition in parrotfish (Scarus schlegeli) and triggerfish (Rhinecanthus aculeatus). Journal of experimental biology, 205(9), 1253-1263. |
| *Schizothorax wangchiachii* | Cyrpiniformes | Cyprinidae | BP | 2.87 | 8.59 | 25.0 | 207.1 | Whilst swimming | No | Yan, G. J., He, X. K., Cao, Z. D., & Fu, S. J. (2013). An interspecific comparison between morphology and swimming performance in cyprinids. Journal of evolutionary biology, 26(8), 1802-1815. |
| *Scolopsis bilineata* | Perciformes | Nemipteridae | BP | 34.18 | 97.00 | 28.0 | 207.2 | Post exhaustion | Yes | Roche, D. G., Binning, S. A., Bosiger, Y., Johansen, J. L., & Rummer, J. L. (2013). Finding the best estimates of metabolic rates in a coral reef fish. The Journal of experimental biology, 216(11), 2103-2110. |
| *Scolopsis bilineata* | Perciformes | Nemipteridae | BP | 44.58 | 97.00 | 28.0 | 270.2 | Whilst swimming | Yes | Roche, D. G., Binning, S. A., Bosiger, Y., Johansen, J. L., & Rummer, J. L. (2013). Finding the best estimates of metabolic rates in a coral reef fish. The Journal of experimental biology, 216(11), 2103-2110. |
| *Scomber japonicus* | Perciformes | Scombridae | P | 992.80 | 1000.00 | 24.0 | 801.2 | Whilst swimming | No | Sepulveda, C. H. U. G. E. Y., & Dickson, K. A. (2000). Maximum sustainable speeds and cost of swimming in juvenile kawakawa tuna (Euthynnus affinis) and chub mackerel (Scomber japonicus). Journal of Experimental Biology, 203(20), 3089-3101. |
| *Scomber scombrus* | Perciformes | Scombridae | P | 546.39 | 1150.00 | 17.0 | 561.4 | Whilst swimming | No | Killen unpublished |
| *Scophthalmus maximus* | Pleuronectiformes | Scophthalmidae | B | 108.86 | 500.00 | 18.0 | 235.1 | Post exhaustion | No | Mallekh, R.&Lagardère, J. P. (2002). Effect of temperature and dissolved oxygen concentration on the metabolic rate of the turbot and the relationship between metabolic scope and feeding demand. Journal of Fish Biology 60, 1105–1115. doi: 10.1006/jfbi.2002.1918 |
| *Seriola lalandi* | Perciformes | Carangidae | P | 1311.60 | 2000.00 | 20.0 | 676.1 | Whilst swimming | No | Clark, T. D., & Seymour, R. S. (2006). Cardiorespiratory physiology and swimming energetics of a high-energy-demand teleost, the yellowtail kingfish (Seriola lalandi). Journal of Experimental Biology, 209(19), 3940-3951. |
| *Silurus meridionalis* | Siluriformes | Siluridae | B | 17.42 | 43.60 | 27.5 | 233.0 | Post exhaustion | Yes | Fu, S. J., Xie, X. J., & Cao, Z. D. (2005). Effect of fasting on resting metabolic rate and postprandial metabolic response in Silurus meridionalis. Journal of Fish Biology, 67(1), 279-285. |
| *Silurus meridionalis* | Siluriformes | Siluridae | B | 3.55 | 21.92 | 21.0 | 129.8 | Whilst swimming | Yes | Pang, X., Cao, Z.-D., Peng, J.-L. & Fu, S. J. (2010). The effects of feeding on the swimming performnce and metabolic response of juvenile southern catfish, Silurus meridionalis, acclimated at different temperatures. Comparative Biochemistry and Physiology, Part A 155, 253-258. |
| *Solea solea* | Pleuronectiformes | Solidae | B | 23.85 | 150.00 | 19.7 | 148.6 | Post exhaustion | No | Lefrancois, C. & Claireaux, G. (2003). Influence of ambient oxygenation and temperature on metabolic scope and scope for heart rate in the common sole Solea solea. Marine Ecology Progress Series 259, 273–284. |
| *Spinibarbus sinensis* | Cypriniformes | Cyprinidae | BP | 0.57 | 4.80 | 25.0 | 71.6 | Whilst swimming | No | Yan, G. J., He, X. K., Cao, Z. D., & Fu, S. J. (2013). An interspecific comparison between morphology and swimming performance in cyprinids. Journal of evolutionary biology, 26(8), 1802-1815. |
| *Stethojulis bandanensis* | Perciformes | Labridae | BP | 83.95 | 15.80 | 27.7 | 2931.3 | Whilst swimming | No | Fulton, C. J., Johansen, J. L.&Steffensen, J. F. (2013). Energetic extremes in aquatic locomotion by coral reef fishes. PLoS One 8, e54033. doi: 10.1371/journal.pone.0054033 |
| *Thunnus albacares* | Perciformes | Scombridae | P | 1939.41 | 1246.67 | 23.0 | 1337.5 | Whilst swimming | No | Dewar, H., & Graham, J. (1994). Studies of tropical tuna swimming performance in a large water tunnel-energetics. The Journal of experimental biology, 192(1), 13-31. |
| *Thunnus orientalis* | Perciformes | Scombridae | P | 4233.00 | 8500.00 | 20.0 | 547.1 | Whilst swimming | No | Blank, J. M., Morrissette, J. M., Farwell, C. J., Price, M., Schallert, R. J., & Block, B. A. (2007). Temperature effects on metabolic rate of juvenile Pacific bluefin tuna Thunnus orientalis. Journal of Experimental Biology, 210(23), 4254-4261. |
| *Zacco platypus* | Cyrpiniformes | Cyprinidae | BP | 3.92 | 10.75 | 25.0 | 228.9 | Whilst swimming | No | Yan, G. J., He, X. K., Cao, Z. D., & Fu, S. J. (2013). An interspecific comparison between morphology and swimming performance in cyprinids. Journal of evolutionary biology, 26(8), 1802-1815. |
|  |  |  |  |  |  |  |  |  |  |  |
| ***AVERAGED DATA FOR G. MORHUA IN FIGURE 2:*** | |  |  |  |  |  |  |  |  |  |
| *Gadus morhua* | Gadiformes | Gadidae | BP | 44.387 | 188.4 | 10 | 374.24 | Post exhaustion |  | Hanna, S. K., Haukenes, A. H., Foy, R. J., & Buck, C. L. (2008). Temperature effects on metabolic rate, swimming performance and condition of Pacific cod Gadus macrocephalus Tilesius. Journal of Fish Biology, 72(4), 1068-1078. |
| *Gadus morhua* | Gadiformes | Gadidae | BP | 198.8 | 1000.0 | 7 | 399.07 | Post exhaustion |  | Jordan, A. D. & Steffensen, J. F. (2007). Effect of ration size and hypoxia on specific dynamic action in the cod. Physiological and Biochemical Zoology 80, 178–185. |
| *Gadus morhua* | Gadiformes | Gadidae | BP | 183.07 | 1000.0 | 5 | 409.09 | Post exhaustion |  | Lapointe, D., H. Guderley, and J.-D. Dutil. (2006) Changes in condition factor have an impact on metabolic rate and swimming performance relationships in Atlantic cos (Gadus morhua L.). Physiological and Biochemical Zoology 79: 109-119. |
| *Gadus morhua* | Gadiformes | Gadidae | BP | 214.87 | 1000.0 | 5 | 480.14 | Post exhaustion |  | Reidy, S. P., Nelson, J. A., Tang, Y.&Kerr, S. R. (1995). Post-exercise metabolic rate in Atlantic cod and its dependence upon the method of exhaustion. Journal of Fish Biology 47, 377–386. |
| *Gadus morhua* | Gadiformes | Gadidae | BP | 74.292 | 299.0 | 15 | 308.11 | Post exhaustion |  | Reidy, S. P., Nelson, J. A., Tang, Y.&Kerr, S. R. (1995). Post-exercise metabolic rate in Atlantic cod and its dependence upon the method of exhaustion. Journal of Fish Biology 47, 377–386. |
| *Gadus morhua* | Gadiformes | Gadidae | BP | 58.967 | 150.0 | 15 | 472.87 | Post exhaustion |  | Schurmann, H., and J. F. Steffensen. 1997. Effects of temperature, hypoxia and activity on the metabolism of juvenile Atlantic cod. Journal of Fish Biology 50: 1166-1180. |
| *Gadus morhua* | Gadiformes | Gadidae | BP | 193.51 | 1000.0 | 11 | 313.49 | Post exhaustion |  | Soofiani, N. M., & Priede, I. G. (1985). Aerobic metabolic scope and swimming performance in juvenile cod, Gadus morhua L. Journal of Fish Biology, 26(2), 127-138. |
| *Gadus morhua* | Gadiformes | Gadidae | BP | 114.53 | 650.0 | 10 | 295.52 | Post exhaustion |  | Sylvestre, E.-L., Lapointe, D., Dutil, J.-D. & Guderley, H. (2007). Thermal sensitivity of metabolic rates and swimming performance in two latitudinal separated populations of cod, Gadus morhua L. Journal of Comparative Physiology B 177, 447–460. doi: 10.1007/s00360-007-0143-x |
| *Gadus morhua* | Gadiformes | Gadidae | BP | 207.08 | 1000.0 | 7 | 415.69 | Whilst swimming |  | Tirsgaard, B., Behrens, J.W. & Steffensen, J. F. (2015). The effect of temperature and body size on metabolic scope for activity in juvenile Atlantic cod Gadus morhua L. Comparative Biochemistry and Physiology A 179, 89–94. |
| *Gadus morhua* | Gadiformes | Gadidae | BP | 147.24 | 1000.0 | 5 | 329.03 | Whilst swimming |  | Lapointe, D., H. Guderley, and J.-D. Dutil. (2006) Changes in condition factor have an impact on metabolic rate and swimming performance relationships in Atlantic cos (Gadus morhua L.). Physiological and Biochemical Zoology 79: 109-119. |
| *Gadus morhua* | Gadiformes | Gadidae | BP | 207.13 | 1000.0 | 11 | 335.56 | Whilst swimming |  | Reidy, S. P., Nelson, J. A., Tang, Y.&Kerr, S. R. (1995). Post-exercise metabolic rate in Atlantic cod and its dependence upon the method of exhaustion. Journal of Fish Biology 47, 377–386. |
