# Supplementary material for: Do method and species lifestyle affect measures of maximum metabolic rate in fishes?
Source: J Fish Biol. 2016 Oct 25;90(3):1037–46. doi: 10.1111/jfb.13195 (PMC5347950; doi:10.1111/jfb.13195)
Supplement: Supplementary file 2 — fig. S1. Phylogenetic tree of the relationships between fish species included in the present study. Phylogenetically informed analyses were performed using the phylogenetic generalized least squares (PGLS) method (Grafen, 1989; Martins & Hansen, 1997; Garland & Ives, 2000) was employed via the ape package (Paradis et al., 2004) in R ( www.r‐project.org), applying a phylogeny generated from the comprehensive tree of life (Hinchliff et al., 2015) using the rotl package (Michonneau et al., 2016), which was then manually augmented. The branch lengths were estimated using Grafen's branch‐length transformation (Grafen, 1989) (branch lengths set to a length equal to the number of descendant tips minus one). The effect of method for estimating maximum metabolic rate (R MMR; swimming or post‐exercise method) was non‐significant whether the PGLS model included lifestyle (effect of method without lifestyle: t = −0·11, P > 0·05; effect of method with lifestyle included in model: t = 0·149, P > 0·05). [file JFB-90-1037-s002.docx]

**FIG. S1.** Phylogenetic tree of the relationships between fish species included in the present study. Phylogenetically informed analyses were performed using the phylogenetic generalised least squares (PGLS) method ([Grafen, 1989](#_ENREF_2); [Martins & Hansen, 1997](#_ENREF_4); [Garland & Ives, 2000](#_ENREF_1)) was employed *via* the ape package ([Paradis *et al.*, 2004](#_ENREF_6)) in R (www.r-project.org), applying a phylogeny generated from the comprehensive tree of life ([Hinchliff *et al.*, 2015](#_ENREF_3)) using the rot’ package ([Michonneau *et al.*, 2016](#_ENREF_5)), which was then manually augmented. The branch lengths were estimated using Grafen’s branch-length transformation ([Grafen, 1989](#_ENREF_2)) (branch lengths set to a length equal to the number of descendant tips minus one). The effect of method for estimating maximum metabolic rate (*R*_MM_; swimming or post-exercise method) was non-significant whether or not the PGLS model included lifestyle (effect of method without lifestyle: *t* = –0.11, *P* > 0.05; effect of method with lifestyle included in model: *t* = 0.149, *P* > 0.05).

**Literature Cited**

Garland, T., Jr. & Ives, A. R. (2000). Using the past to predict the present: Confidence intervals for regression equations in phylogenetic comparative methods. *American Naturalist* **155**, 346-364.

Grafen, A. (1989). The phylogenetic regression. *Philosophical Transactions of the Royal Society of London B* **326**, 119-157.

Hinchliff, C. E., Smith, S. A., Allman, J. F., Burleigh, J. G., Chaudhary, R., Coghill, L. M., Crandall, K. A., Deng, J., Drew, B. T. & Gazis, R. (2015). Synthesis of phylogeny and taxonomy into a comprehensive tree of life. *Proceedings of the National Academy of Sciences* **112**, 12764-12769.

Martins, E. P. & Hansen, T. F. (1997). Phylogenies and the comparative method: A general approach to incorporating phylogenetic information into the analysis of interspecific data. *American Naturalist* **149**, 646-667.

Michonneau, F., Brown, J. & Winter, D. (2016). rotl, an R package to interact with the Open Tree of Life data. *Peer J Preprints* **4**, e1471v1473.

Paradis, E., Claude, J. & Strimmer, K. (2004). APE: Analyses of Phylogenetics and Evolution in R language. *Bioinformatics* **20**, 289-290.
